# Emerging concepts of reactive oxygen species functions in plants

**DOI:** 10.1042/BST20250107

**Published:** 2026-04-29

**Authors:** Christine H. Foyer

**Affiliations:** School of Biosciences, College of Life and Environmental Sciences, University of Birmingham, Edgbaston B15 2TT, U.K.

**Keywords:** hydrogen peroxide, nucleus, oxidation-reduction, post translational modification, respiratory burst oxidase homologs, superoxide

## Abstract

Reactive oxygen species (ROS) are ubiquitous signalling molecules that serve to integrate developmental, metabolic and stress signals to shape adaptive outcomes, linking energy metabolism to plant physiology, growth and stress resilience in a changing world. Resolving the factors and mechanisms involved in ROS-mediated control has proved to be far from trivial, not least because ROS are produced by every compartment of plant cells, serving multiple functions with numerous points of reciprocal control between phytohormones and other signalling pathways. While many enzymes produce hydrogen peroxide (H_2_O_2_) directly, other ROS sources such as respiratory burst oxidase homologues (RBOH) produce superoxide as a primary product. Key questions remain concerning the respective roles of superoxide and H_2_O_2_ in redox regulation of plant growth, development and defence, and how plant cells can differentiate between ROS produced in different cellular compartments. One solution concerns cysteine (Cys) molecular switches, which are specialised protein thiols that operate as highly sensitive ROS sensors in different locations, transducing changes in oxidation status to the nucleus and facilitating functional changes in protein activity, structure, and localisation. In addition, it is likely that the localisation and positions of many redox proteins, such as catalase and RBOH, are not as fixed as initially proposed, allowing plasticity of function in different compartments. This review discusses current concepts in plant ROS biology, highlighting novel aspects that permeate every aspect of plant biology.

## Introduction

The origin story of the identification of the production of reactive oxygen species (ROS) in plants began with the characterisation of the Mehler reaction [1] in photosynthesis. In this process, ground state molecular oxygen is reduced to H_2_O_2_, by the photosynthetic electron transport chain (PETC), primarily on the reducing side (acceptor side) of the Photosystem I (PSI) complex [2,3]. Thereafter, it was shown that the superoxide radical is the primary product of this reaction, from which H_2_O_2_ is produced by the action of superoxide dismutase [4]. While there are many potential sites of superoxide production in the PETC, including photosystem II and ferredoxin, O_2_ reduction mainly occurs in the distal iron–sulfur clusters and the phylloquinone moiety of the PSI complex [5]. The concept that chloroplasts not only use ROS to regulate chloroplast metabolism but also as signals to modulate plastid and nuclear gene expression came much later [3]. It is now generally accepted that ROS production provides a critical mechanism by which plants monitor the functional state of the photosynthetic machinery and adjust gene expression in the nucleus to adapt to environmental fluctuations [2,3].

After the initial discovery in chloroplasts, ROS production in mitochondria was established. This process involves both one-electron transfers to oxygen to form superoxide or two-electron reductions to form H_2_O_2_ [6]. Mitochondria generate superoxide and/or H_2_O_2_, either in the matrix or on the cytosolic side of the inner membrane. As with chloroplasts, mitochondrial ROS signals participate in plant mitochondrial-to-nucleus communication, a process known as mitochondrial retrograde regulation (MRR), to relay information concerning mitochondrial dysfunction to the nucleus [7]. ROS produced within mitochondria can be transferred to the cytosol through voltage-dependent anion-selective channel proteins (VDACs) that are localised to the outer mitochondrial membrane, forming a key gateway for ROS movement [8].

**Figure 1:**
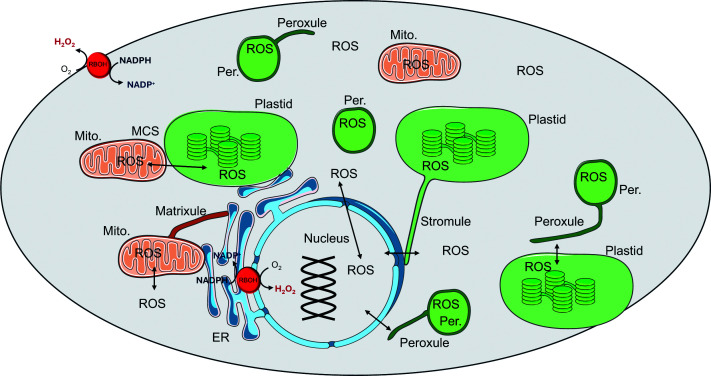
Diagrammatic representation of the different sites of production of reactive oxygen species (ROS) production in plant cells, together with an indication of ROS transfer between compartments. ER: endoplasmic reticulum; Mito: mitochondria; Per; peroxisome; RBOH: respiratory burst oxidase homologue ROS are essential metabolites that are produced in large amounts in all the compartments of plant cells. ROS can move between compartments via transport proteins such as PIPs, or through organelle–organelle interactions. Mitochondria, plastids and peroxisomes form tubular structures: stromules in plastids, matrixules in mitochondria and peroxules in peroxisomes. These tubular structures can mediate communication between organelles by exchanging signalling molecules and metabolites. RBOH: respiratory burst oxidase homologue.

Superoxide production by the outer surface of the plant plasma membrane in what is now known as the “oxidative burst” was identified through the pioneering work of Noriyuki Doke [9]. A primary source of the plasma membrane-localised oxidative burst was thereafter shown to be the respiratory burst oxidase homologues (RBOH) enzymes, which are plant-specific NADPH oxidases that are often considered to be the primary source of ROS signals, generating ROS signals under tight spatiotemporal control [10]. Class III peroxidases in the apoplast/cell wall compartment were later shown to produce H_2_O_2_ and to contribute to the pathogen-induced oxidative burst. Such peroxidases can act independently of RBOH to produce rapid ROS bursts upon perception of pathogens. In addition, many other metabolic enzymes were found to generate superoxide and H_2_O_2_. Hence, rather than “by-products”, of metabolism, a wealth of literature shows that ROS are true products of plant metabolism, generated, for example through the action of the enzyme glycollate oxidase in photorespiration. Moreover, ROS such as H_2_O_2_, are not confined to the production sites, but they can move between different compartments via channels such as the specific group of aquaporins called plasma membrane intrinsic proteins (PIPs), and possibly also through contact sites between compartments, as illustrated in Figure 1.

Like ROS production, redox regulation of proteins mediated by thiol-disulfide exchange reactions and driven by the transmission of reductive and oxidative signals from the PETC to target enzymes of the Calvin–Benson cycle (CBC) such as fructose-1,6-bisphosphatase (FBPase) was first discovered and established in chloroplasts [11]. The reversible reductive activation of proteins is mediated by the flux of electrons from Fd to thioredoxins (Trxs) via Fd-Trx reductase, or through Fd-NADP^+^ reductase and NADPH-dependent Trx reductase C [12]. This redox cascade activates carbon assimilation in response to light by activating key CBC enzymes, while high-midpoint-potential atypical Trxs, such as Trx-like 2 (TrxL2), atypical Cys His-rich thioredoxin (ACHT), and Trx-*f* act as oxidising factors that inactivate the CBC enzymes in the dark, working together with the FTR and the NTRC systems to donate electrons to 2-Cys peroxiredoxin (2-Cys Prx) with H_2_O_2_ as the final acceptor [13–15]. Low molecular weight antioxidants such as ascorbate and glutathione and antioxidant enzymes such as catalase (CAT) function alongside this network as crucial redox guardians that serve to police redox signals, having interactions with multiple regulatory and signalling pathways [16].

Plants contain an extensive and diverse array of redox proteins that, together with low-molecular-weight antioxidants and antioxidant enzymes [16,17], form a flexible multi-layered redox network that integrates metabolic and environmental signals to optimise plant functions. Plants possess over 20 types of Trxs that are distributed across cellular compartments, including chloroplasts, mitochondria, the cytosol, and the nucleus [18]. Together with the 2-Cys Prx and other redoxins, such as glutaredoxins (GRX), the Trx proteins not only maintain redox homeostasis, but they also serve as crucial signalling functions by modulating redox post-translational modifications (PTMs) on proteins and transcription factors that control cell functions and fate. The goals of this review are to provide a brief overview of current concepts and emerging facets of ROS functions in plants, summarising regulatory mechanisms and compartment-specific pathways of redox signalling.

## ROS functions and signalling pathways

The concept that ROS are harmful to cells is attributed to Rebeca Gershman in 1954, who proposed the “Free Radical Theory of Oxygen Toxicity”. This theory, which laid the foundations of concepts of ROS-induced cellular damage, was followed and expanded in 1956 by Denham Harman’s “Free Radical Theory of Aging,” which proposed that ROS derived from metabolism are the primary cause of ageing and damage to cellular macromolecules. The discovery of superoxide dismutase (SOD) by McCord and Fridovich in 1969 [6] provided direct evidence of an enzyme designed to speed up the removal of superoxide radicals, while somewhat ignoring the fact that SOD simply converts one ROS form into another. While the Free Radical Theory of Aging is no longer accepted in its original form, alongside the notion that antioxidants are a “Fountain of Youth”, the attractive simplicity of the Free Radical Theory of Oxygen Toxicity has persisted as a dominant paradigm [19], despite evidence that most ROS forms have only limited reactivity, as discussed below, and the emergence of more modern concepts concerning thiol switches and integration of redox regulation on multiple levels. The often-stated view that low levels of ROS act as signals while high levels are toxic is outdated in the plant context, or, at best, it is a limited component of the more complex ROS roles in the triggering of genetically controlled cell suicide programmes.

The reactivity of ROS, particularly H_2_O_2_, dictates that they are excellent signalling molecules. However, the view that ROS are harmful species that cause oxidative damage to proteins, lipids and nucleic acids remains firmly embedded in the literature [19]. The limited chemistry of superoxide and H_2_O_2_ would prevent direct interactions with any of these molecules. In fact, while the reactivity of superoxide is greater than that of ground-state molecular oxygen, it is low compared with other reactive species [20]. With its low mobility and a short half-life, the actual reactivity of superoxide in biological systems is more nuanced than other ROS. It can reduce both ascorbate and the oxidised ferric iron (Fe^3+^) forms to ferrous iron (Fe^2+^). When this occurs in the Fe moieties of plant proteins, it can have significant consequences for protein functions. Superoxide can release Fe^2+^ from [Fe-S] proteins and from ferritin, and it can also produce nitric oxide (NO) radical to produce peroxynitrite. H_2_O_2_ has more limited reactivity than superoxide in terms of directly reacting with biological molecules. It is generally poor at directly oxidising common cellular components such as DNA, lipids, and most proteins, and so it is considered a relatively mild and selective oxidant in biological systems, which is consistent with its role as a signalling molecule. Its reactivity is limited to specific target amino acids with low pKa values, most notably Cys thiol groups. Cysteine (Cys) plays structural and regulatory roles in plant proteins and in glutathione, contributing to maintaining redox homeostasis and regulating signalling within and amongst cells. The selective and reversible oxidation of protein Cys residues is fundamental to cell signalling, regulating a wide range of plant processes from cell proliferation and differentiation to plant development and defence [20–23].

Starting from the reduced form (SH), the sulfur atom of a Cys residue can undergo a wide range of oxidative modifications. This reactivity is greatly enhanced for Cys, whose thiol side chain is in the thiolate form, i.e., deprotonated at physiological pH (S^−^), and is influenced by structural factors. The oxidation of Cys thiols results initially in the formation of a highly unstable sulfenic acid (–SOH), which can be further oxidised to sulfinic acid (–SO_2_H) or sulfonic acid (–SO_3_H), or can react with other Cys residues to form disulfide bonds ([24], see for example Figure 1 in [25]). Reactive nitrogen species (RNS) such as NO react with some Cys residues, resulting in S-nitrosylation/nitrosation. Moreover, some Cys residues can also undergo lipid modifications such as palmitoylation and prenylation, or they can bind metals such as Zn, Fe and Cu, for example in the formation of zinc fingers and iron–sulfur clusters.

The plant sulfenome refers to the comprehensive inventory of protein Cys residues that undergo reversible oxidation to sulfenic acid. These are an integral part of redox networks and are linked by kinetically controlled redox switches within the proteome, in which protein conformation, macromolecular interactions, trafficking through thiol-disulfide protein structures, activity, and function play an essential role. Like protein S-sulfenylation, thiol-disulfide exchange is a critical PTM of Cys residues that participates in redox signalling and protein regulation. However, while S-sulfenylation acts as an oxidative switch, thiol**-**disulfide exchange involves the rearrangement or formation of disulfides, resulting in no net change in the oxidation state of the system.

The oxidation of Cys thiols forms the basis for ROS receptor and redox switch functions in different cellular compartments. Instead of a single receptor, plants sense ROS through a diverse range of proteins, many of which remain to be unidentified. The network of plant redoxins and thiol peroxidases encompasses many redox switches, which are involved in redox sensing and signal transduction, translating metabolic environmental cues into developmental and physiological responses. The plasma membrane-localised leucine-rich repeat receptor-like kinase called HPCA1 (Hydrogen Peroxide-Induced Calcium Increase 1) and CANNOT RESPOND TO DMBQ 1 are of particular importance in local and systemic cell-to-cell signalling [26]. HPCA1 functions as a direct apoplastic H_2_O_2_ and quinone sensor that mediates and coordinates the systemic cell-to-cell ROS and calcium signals that mediate plant stress responses [27]. Such receptors operate together with the aquaporin subfamily of PIPs, which facilitate H_2_O_2_ transport across plant cell membranes [28].

Redox modifications of protein Cys residues enable the regulation of the interactome of a given protein. For example, the accumulation of ROS in root nuclei triggers the redox-dependent multimerisation of the auxin repressor protein IAA3, a process that facilitates interactions with the co-repressor called Topless, thereby attenuating IAA3-mediated target gene repression that shapes root adaptive responses [29]. Interestingly, the accumulation of ROS in the nuclei that accompanies this regulation is dependent on the activities of RBOH enzymes [29]. Similarly, guanylate binding protein-like GTPases undergo phase transition to regulate transcriptional responses involved in plant immunity [30]. Another important transcription factor that regulates systemic acquired immunity, the EXPRESSOR OF PATHOGENESIS-RELATED GENES 1, also forms condensates to regulate plant cell death responses [31]. Such redox PTMs are site-specific and governed by the microenvironment of the Cys residues, where they are subject to temporal and spatial controls.

Oxidative modifications of protein Cys residues are also a key driver of protein phase separation, acting as a redox-sensitive switch that promotes the formation of biomolecular condensates. For example, Cys oxidation drives a phase separation transition in the terminating flower transcription factor in tomato plants, a process that allows binding to the promoter of the floral identity gene *ANANTHA* [32]. The redox regulation of protein condensates in plants is thus an emerging mechanism for stress response and developmental control, where the cellular redox state acts as a switch for the assembly, disassembly, or localisation of biomolecular condensates. The fluidity and interaction dynamics of such membrane-less organelles are often regulated by the redox state of Cys residues, conferring the ability to concentrate specific proteins and RNAs in response to environmental cues, such as heat stress or pathogen infection [33]. This regulation can also lead to the formation of redox-sensitive protein composites, such as disulfide-linked dimers and the assembly of higher-order protein structures such as stress granules [34].

**Figure 2:**
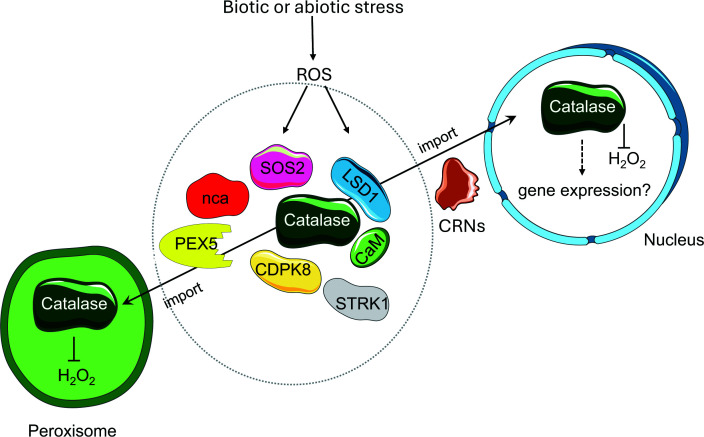
Diagrammatic representation of different proteins that can bind to the catalase protein in the cytosol and influence function and/or intracellular localisation. CaM: calmodulin; CPKD8: calcium dependent protein kinase 8; CRNs: Crinkling and Necrosis effectors LSD1: LESION SIMULATING DISEASE1; nca: NO CATALASE ACTIVITY1; PEX5: peroxin 5; SOS2: a class 3 sucrose-nonfermenting 1-related kinase; STRK1: Salt Tolerance Receptor-Like Cytoplasmic Kinase 1 Redox regulation of catalase interactions with LSD1 and other binding proteins in the cytosol can regulate intracellular localisation and activity. CaM: calmodulin; CDPK8: calcium-dependent protein kinase 8; SOS2: salt overly sensitive 2; LSD1: lesion simulating disease 1; STRK1: receptor-like cytoplasmic kinase; nca: no catalase activity 1; and PEX5: a plant peroxisomal import protein.

The oxidisation of protein Cys residues and redox posttranslational modifications are also important in the regulation of autophagy [35] and programmed cell death (PCD). The extent and duration of ROS accumulation determine cell fate in terms of either improved vigour and sustainability, or death through genetically determined suicide programme termed PCD. PCD is not only important for animal and plant development but also for appropriate responses to biotic and abiotic stresses, all of which trigger enhanced ROS production. However, the redox regulation of these pathways is mediated by CAT as well as ROS. Plant CATs interact with a variety of integral stress signalling proteins in the cytosol (Figure 2), including calmodulin, calcium-dependent protein kinase 8, salt overly sensitive 2, lesion simulating disease 1 (LSD1), receptor-like cytoplasmic kinase STRK1, and no catalase activity 1 [36]. CAT is also a target for pathogen-encoded effector proteins [37,38]. The fungal effectors PsCRN115 and PsCRN63 traffic CAT to the nucleus, but while PsCRN115 stabilises CAT, leading to decreased accumulation of H_2_O_2_, and lower PCD, binding to PsCRN63 destabilises CAT, increasing H_2_O_2_ and PCD [39]. With some exceptions (apoplast and endoplasmic reticulum), most plant cells compartments are rich in low-molecular-weight antioxidants and antioxidant enzymes, which have a significant capacity to relocate in response to appropriate signals.

Several proteins in the conserved autophagy pathway, such as ATG4 activity, are regulated by H_2_O_2_-mediated oxidation of specific Cys residues, which are considered to act as a “redox switch” that modulates the autophagy pathway, particularly during starvation [40]. The *Arabidopsis* CAT2 protein is recruited to phase-separated condensates with LSD1, which is a plant-specific PCD regulator, in a redox-dependent manner that regulates its intracellular localisation and activity [41].

## Cell proliferation, growth, and development

RBOH-derived ROS drive plant growth, meristem maintenance, and cellular proliferation [42]. Phytohormones such as auxin and abscisic acid trigger the activation of RBOH [43]. For example, changes in root system architecture upon perception of dry air spaces in the soil involve the ROS-dependent oxidation of the auxin repressor protein IAA3 [29]. Oscillating levels of ROS produced by RbohC and RbohE occur within the *Arabidopsis* root cell cap. These changes are negatively regulated by the NAC transcription factor ANAC33/SOMBRERO [44]. The cells in the lateral root cap undergo PCD, while the outermost columella root cap cells become detached. The oscillating ROS levels that occur within the columella during development are required for the initial division and detachment within the columella. They also affect auxin distribution by modulating the transporter PIN-FORMED2 [44].

While functional redundancy is a common feature of plant RBOHs, accumulating evidence suggests that these enzymes play a crucial role in plant growth and development. RBOH activity is controlled by Ca^2+^ binding, and by a range of PTMs, including protein phosphorylation, nitrosylation, persulfidation, and ubiquitination [45,46]. Persulfidation of RBOHD is important in the ABA-mediated control of stomatal opening in guard cells [47]. Regulation by Ca^2+^-regulated protein kinases and Ca^2+^ binding to EF-hand motifs is a ubiquitous mechanism of RBOH activity regulation. Waves of ROS and Ca^2+^ signals work together through the coordination and integration of apoplastic and symplastic signalling pathways [48], which in turn trigger MITOGEN-ACTIVATED PROTEIN KINASE signalling cascades and other similar pathways that regulate nuclear gene expression. The regulation of RBOH activity is triggered primarily through interactions with membrane-bound kinases and signalling proteins that act downstream of surface receptor complexes, such as Pattern Recognition Receptors [42]. For example, the phosphorylation of RBOHD BOTRYTIS-INDUCED KINASE 1 is essential for Ca^2+^-based activation of RBOHD activity upon pathogen perception [49]. The serine-threonine protein phosphatases inhibit RBOH activity. Similarly, the SnRK2-type kinase OPEN STOMATA1 is activated by ABA, leading to the phosphorylation of RBOHF and stomatal closure [50].

Redox PTMs are likely to play a key role in cell proliferation [51,52]. The current model proposes that there is a transient oxidation event early in the G1 phase of the cell cycle, which is required for cells to progress into the S phase. This process is part of a “redox cycle” in which ROS levels and antioxidants such as reduced glutathione (GSH) oscillate to regulate the activity of cell-cycle regulatory proteins [53–55]. GSH availability and ROS patterning regulate the cell cycle in the root apical meristem (RAM), as well as root development and growth [56]. GSH is enriched in the nucleus of proliferating cells in both plants and animals [57]. GSH is also required for root growth [58], playing a critical role in maintaining RSA by controlling meristem activity, cell proliferation, and root hair development [59]. Injury to the RAM results in ROS accumulation, with cells in G1 undergoing a transient peak in the GSH level in the nucleus prior to coordinated entry into S phase followed by rapid divisions and cellular reprogramming [60].

An increasing number of protein targets for ROS-mediated PTMs have recently been identified in animal cell proliferation, including cyclin-dependent kinase (CDK) [61], CDK inhibitors [62], and the TFIIB-related factor 2 transcription factor [63]. Direct targets of ROS-mediated modification in plant growth and development include mitogen-activated protein kinases [63], and transcription factors such as TEOSINTE BRANCHED1 and CYCLOIDEA [64,65] and BRASSINAZOLE-RESISTANT/BRI1-EMS-SUPPRESSOR [66]. In addition, several key meristem regulators have been shown to function downstream of ROS signalling [67–70].

The balance of O_2_^•−^ and H_2_O_2_ is regulated in the transition zone of the developing root [63]. Cell division is terminated and elongation begins at the point where H_2_O_2_ levels exceed superoxide accumulation [69]. The balance between superoxide and H_2_O_2_ in the root is determined by the UPBEAT1 (UPB1) transcription factor, which modulates the expression of peroxidases and the switch from cell proliferation to expansion and differentiation [67]. The UPB1 transcription factor is also expressed in the shoot apical meristem (SAM). The *upb1-1* mutants have a smaller SAM, but with a similar stem cell region. Moreover, higher peroxidases but lower levels of H_2_O_2_ were detected in the SAM of the *upb1-1* mutants [68]. Such findings clearly indicate that superoxide and H_2_O_2_ serve different regulatory and signalling functions in the developing roots and shoots, with superoxide signalling through control of DNA methylation [70] and protein Fe moieties, and H_2_O_2_ functions through PTMs of key proteins.

## Perspectives

- ROS are essential, dynamic regulators of plant cell functions and fate, influencing every aspect of plant biology. Through interactions with a wide range of proteins and other molecules, ROS regulate metabolism, phytohormone synthesis, transport and signalling, immune and developmental controls and their responses to environmental stress. Crosstalk between the ROS and phytohormone signalling pathways, such as auxin and salicylic acid, is a central axis of plant growth, development and immunity, and there are multiple points of reciprocal control. Functioning alongside superoxide and singlet oxygen signalling pathways, redox PTMs provide a reversible switching mechanism that is responsible for a wide range of genetic and epigenetic controls.
- Current thinking concurs that superoxide and H_2_O_2_ serve as regulatory and signalling molecules in plants, functioning through pathways, particularly redox PTMs. Even the hydroxyl radical, which is often considered to be the most reactive and short-lived ROS, is now generally not considered to be an agent of destruction in plants but rather an important component of plant growth and stress responses [71]. ROS themselves do not serve as cytotoxic agents, as has often been suggested in the literature; rather, regulated ROS accumulation, which is generally linked to regulated decreases in antioxidant defences, activates genetically controlled cell suicide programmes and PCD. The sensitivity of cells to ROS-regulated adaptation or PCD responses is dictated by cell identity. For example, high ROS levels define the stem cell niche. The perception of different ROS forms is enabled by a wide range of ROS receptor molecules, which are subject to redox regulation and/or PTMs, in each compartment of the cell, including the nucleus.
- We still only have a basic understanding of the nature and functions of redox sensors in different plant cell compartments, and the pathways that transmit information from the different compartments to the nucleus. Crucially, little is known about the ability of the nucleus to generate ROS and how nuclear ROS accumulation directly regulates genetic and epigenetic controls. Redox signalling is tightly controlled by a combination of multiple factors such as local protein environment, intracellular redox compartmentalisation, and enzymatic reduction systems, but our understanding of these factors in different situations and cell types remains poorly defined. In addition, much remains uncertain about how organelle–organelle interactions are regulated, either the formation of tubular structures by one organelle or membrane contact sites and how this is involved in the transfer of ROS or redox signals between different compartments.

## Data Availability

There are no new data associated with this article.

## Open Access

Open access for this article was enabled by the participation of the University of Birmingham in an all-inclusive Read & Publish agreement with Portland Press and the Biochemical Society under an agreement with JISC.

## Abbreviations

2-Cys Prx: 2-Cys peroxiredoxin
ACHT: atypical Cys His-rich thioredoxin
CAT: catalase
CDK: cyclin-dependent kinase
Cys: cysteine
CBC: Calvin–Benson cycle
GRX: glutaredoxins
GSH: glutathione
HPCA1: Hydrogen Peroxide-Induced Calcium Increase 1
LSD1: lesion simulating disease 1
MRR: mitochondrial retrograde regulation
NO: nitric oxide
PTMs: post-translational modifications
PETC: photosynthetic electron transport chain
PIPs: plasma membrane intrinsic proteins
PCD: programmed cell death
PSI: photosystem I
RNS: Reactive nitrogen species
ROS: reactive oxygen species
RBOH: respiratory burst oxidase homologues
RAM: root apical meristem
SAM: shoot apical meristem
SOD: superoxide dismutase
Trxs: thioredoxins
TrxL2: Trx-like 2
UPB1: UPBEAT1
VDACs: voltage-dependent anion-selective channel proteins
